# Distribution of *Oenococcus oeni* populations in natural habitats

**DOI:** 10.1007/s00253-019-09689-z

**Published:** 2019-02-20

**Authors:** Marc P. G. Lorentzen, Patrick M. Lucas

**Affiliations:** Unité de recherche Oenologie, EA 4577, USC 1366 INRA, ISVV, Université de Bordeaux, F-33882 Villenave d’Ornon, France

**Keywords:** *Oenococcus oeni*, Wine, Malolactic fermentation, Genomics, Evolution, Domestication

## Abstract

*Oenococcus oeni* is the lactic acid bacteria species most commonly encountered in wine, where it develops after the alcoholic fermentation and achieves the malolactic fermentation that is needed to improve the quality of most wines. *O. oeni* is abundant in the oenological environment as well as in apple cider and kombucha, whereas it is a minor species in the natural environment. Numerous studies have shown that there is a great diversity of strains in each wine region and in each product or type of wine. Recently, genomic studies have shed new light on the species diversity, population structure, and environmental distribution. They revealed that *O. oeni* has unique genomic features that have contributed to its fast evolution and adaptation to the enological environment. They have also unveiled the phylogenetic diversity and genomic properties of strains that develop in different regions or different products. This review explores the distribution of *O. oeni* and the diversity of strains in natural habitats.

## Introduction

The fate of *Oenococcus oeni* would have been very different if the benefits of performing MLF in wine had not been perceived in the middle of the twentieth century (Davis et al. 1985). *O. oeni* would have been ranked as a minor LAB species barely detectable in the natural environment and more often in fruit juices when they start to ferment. It would also have been considered as a contaminant occurring in wine during aging or storage (Lonvaud-Funel 1999). However since the 1950s, it has been recognized that MLF improves the quality of wine (Davis et al. 1985). MLF has become an essential step for producing all red wines and numerous white wines. In the same time, *O. oeni*, which is the best adapted species in wine, has gained much attention, not only as the key actor in MLF, but also as an industrial product marketed to better control MLF and as one of the most studied LAB species (Bartowsky 2005).

The main transformation that *O. oeni* achieves during MLF is the conversion of l-malate to l-lactate and carbon dioxide, where wine is deacidified and gains a softer taste (Lonvaud-Funel 1999; Versari et al. 1999). MLF lasts a few days, weeks, or months depending on wine making practices. During this period, bacteria metabolize other organic acids, sugars, amino acids, aroma precursors, and diverse compounds. This improves the microbiological stability of wine by removing potential substrates that harmful microorganisms could use to grow, while increasing the aromatic complexity (Davis et al. 1985; Liu 2002; Bartowsky 2005; Sumby et al. 2014).

*O. oeni* is one of three species of *Oenococcus* described to date, but the only one detected in wine. Although wine is its best known environment, *O. oeni* is also a predominant species in other fermented beverages such as cider or kombucha. The first genome sequence showed that *O. oeni* has a rare genetic characteristic: it is hypermutable due to the absence of the DNA mismatch repair system, MutSL, which most likely contributed to its rapid adaptation to the wine environment (Marcobal et al. 2008). There is a great diversity of strains more or less well adapted to wine. Their diversity has long been studied by various molecular methods, although their distribution in different regions and types of wine remained puzzling. Recently, comparative genomics based on genomes of many strains is shedding new light on genetic characteristics, species diversity, and adaptation of *O. oeni* in wines or other habitats (Bartowsky & Borneman 2011; Bartowsky 2017).

## *O. oeni*: the wine LAB

The first strains of *O. oeni* were isolated from wine in the late nineteenth and early twentieth century when it was understood that malic acid was converted to lactic acid and carbon dioxide by wine bacteria during a “secondary fermentation,” which is now called the MLF (Bartowsky 2005). The bacteria were tentatively attributed to species such as *Leuconostoc gracile*, *Bacterium gracile*, *Leuconostoc citrovorum*, or *Leuconostoc mesenteroides* (Bartowsky 2005). In 1967, the species was described for the first time by comparing 19 LAB strains isolated during MLF of wines produced in California, France, and Australia (Garvie 1967). The strains had similar morphological and metabolic characteristics despite being isolated from distant regions, indicating not only that they belonged to the same species, but also that this species predominated during MLF in most wines. The species was named “*Leuconostoc oenos*” owing to phenotypic similarities with *Leuconostoc* species. It is a diplococcus that sometimes forms chains, Gram-positive, microaerophilic, obligatory heterofermentative, producing d-lactate from glucose (along with CO_2_ and ethanol or acetate), acidophilic, and more tolerant to low pH than all other *Leuconostoc* species. In 1995, it was reclassified in a newly created genus “*Oenococcus*” on the basis of molecular analyses that demonstrated its phylogenetical divergence from the genus *Leuconostocs* (Dicks et al. 1995). The first genomic sequence was produced in 2005 from strain PSU-1 (Mills et al. 2005). Although more than 200 genomes are now available, that of PSU-1 has remained the only complete genome published until very recently (Iglesias et al. 2018). It is a rather small genome (1.8 Mb), which has undergone a reductive evolution, losing many biosynthetic pathways for amino acid, vitamins, or cofactors. This denotes a strong specialization for nutrient-rich environments, in agreement with its prevalence in wine. The genome contains only two copies of the rRNA operon, compared to the 4 to 9 copies usually encountered in LAB (Makarova et al. 2006). It is suggested that the rRNA copy number is more important in fast-growing bacteria that require higher translation activity to develop in a fluctuating environment (Klappenbach et al. 2000). In agreement, *O. oeni* is notoriously a slow growth species and it is rarely detected in the natural environment, where it is outcompeted by other species.

## The sister species *O. kitaharae* and *O. alcoholitolerans*

**Fig. 1:**
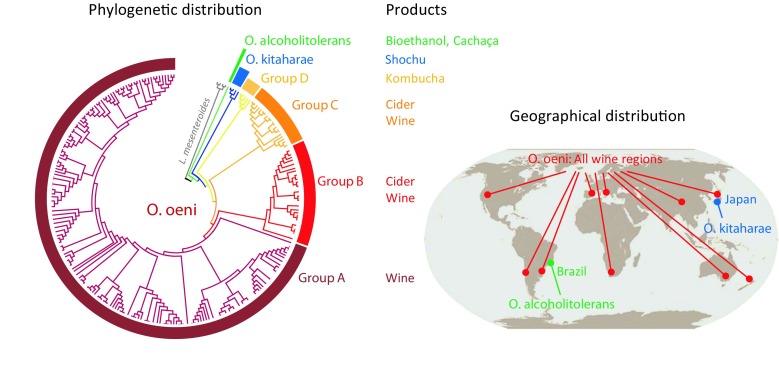
Distribution of *O. oeni*. The figure shows the phylogenomic, geographical, and product type distribution of *O. oeni*, *O. kitaharae*, and *O. alcoholitolerans*. The phylogram is based on genomic distances calculated by average nucleotide identity and plotted with neighbor-joining using 252 whole genome sequences of *O. oeni* strains, 4 *O. kitaharae*, 1 *O. alcoholitolerans*, and 4 *L. mesenteroides* used as outgroup. Phylogenetic groups A, B, C, and D are those defined in Lorentzen et al. (in review)

*O. oeni* has long been the only known representative of the genus *Oenococcus*, although two other species were more recently identified (Fig. 1). In 2006, *Oenococcus kitaharae* was isolated from composting distillation residues of Shochu, a Japanese spirit produced by distillation of fermented rice, sweet potato, barley, and other materials (Endo & Okada 2006). *O. kitaharae* is phylogenetically close from *O. oeni*, but it has different properties such as a higher pH optimum of growth, the inability to convert malic acid into lactic acid and CO_2_, and a different sugar consumption profile (Endo & Okada 2006; Cibrario et al. 2016). Its genome has a similar size as *O. oeni*, with only two sets of rRNA genes and it also lacks the *mutSL* genes, but it contains genetic elements suggesting adaptation to a different environment (Borneman et al. 2012b). *O. kitaharae* carries genes for arginine and histidine biosynthesis, which are rarely present in *O. oeni*, probably because these amino acids are among the most abundant in wine. It has a different repertoire of sugar utilization genes, which correlates with different carbohydrate sources present in wine and in vegetables or cereals used for Shochu production. Orthologues of the 3 genes of the malolactic pathway, which is required for producing MLF, are present in *O. kitaharae*, but a stop codon interrupts prematurely the coding sequence of the malolactic enzyme. This prevents the bacterium from consuming malate and strengthens the idea that it is not adapted to wine. *O. kitaharae* possesses genes for production of bacteriocins and other antimicrobials, a CRISPR system to fight against phages and other defense genes that are hallmarks of a species that develops in a competitive environment where it must fight other microorganisms. These elements are absent or rarely present in *O. oeni*, which has very few competitors in wine (Borneman et al. 2012b). To date, only 6 strains of *O. kitaharae* have been isolated, all from the same sample of composting residues of Shochu (Endo & Okada 2006). The species was presumably detected in Spanish wine (Gonzalez-Arenzana et al. 2013) and Brazilian kefir (Zanirati et al. 2015), but this was not confirmed by isolating strains. On the other hand, *O. oeni* has not been detected in Shochu distillation residues or during its production. Although they are evolutionarily close, it is clear that these two species have evolved to adapt to different environments.

*O. alcoholitolerans* is the third and most recently described species of the genus. The only 4 currently known strains were isolated in 2014 from sugarcane fermentation vats of Brazilian distilleries producing bioethanol and Cachaça (Badotti et al. 2014). Like in Shochu, MLF is not a feature in the sugarcane fermentations; in fact, the LAB are regarded as contaminants in the process (Badotti et al. 2014). A draft genome of 1.2 Mb was assembled of one *O. alcoholitolerans* strain, which showed an almost 25% reduction in coding sequences compared to the other species in the genus. Unlike *O. kitaharae*, the gene of the malolactic enzyme seems to be intact in *O. alcoholitolerans*, although the ability to degrade malate was not verified experimentally. The species is more sensitive to acidity than *O. oeni* and grow at higher ethanol levels than *O. kitaharae* (Badotti et al. 2014). An adaptation to the sugarcane fermentation environment appears to have taken place, as it is able to metabolize sucrose, fructose, and raffinose very well in contrast to *O. oeni*, but has reduced or no ability to degrade maltose, ribose, and trehalose (Badotti et al. 2014; Cibrario et al. 2016).

It is not yet understood why the three *Oenococcus* species are associated with different alcohol-containing environments, but they have different genetic and metabolic properties that favor their predominance in one product over another.

## Wine: the favorite habitat

Wine is undoubtedly the favorite habitat of *O. oeni*. Since the first description of strains isolated from Californian, Australian, and French wines, it has been reported as the predominant species during MLF in wines produced in all regions, at times the only species detected. However, each wine is different and more or less favorable to bacterial growth, which includes the growth of *O. oeni*. It grows better than other LAB because of a superior tolerance to the low pH that is encountered in most wines (typically pH 2.9–3.6) (Davis et al. 1988). However, when pH exceeds 3.4–3.6, *O. oeni* is challenged by various species of *Lactobacilli and Pediococci*, which grow faster and may become predominant and perform MLF (Lonvaud-Funel 1999).

The LAB population in grape must is about 10^2^ to 10^4^ cells mL^−1^ depending on climate conditions and grape quality at harvest time (Lonvaud-Funel 1999). *O. oeni* is only a small part of it. During AF when alcohol content starts to exceed 5 or 6% and becomes a significant stress that adds to that already caused by the low pH, most LAB die and their total population decreases. *O. oeni* resists better and starts to develop towards the end or after AF, when yeast autolysis releases essential nutrients that it needs (Lonvaud-Funel et al. 1991). The degradation of l-malate becomes perceptible when the *O. oeni* population reaches 10^6^ cells mL^−1^. It can increase up to 10^7^–10^8^ cells mL^−1^ until the end of MLF when all malate has been exhausted. *O. oeni* cells are then removed by adding sulfur dioxide and using oenological practices such as decantation, filtration, etc. When sulfur dioxide is not used, the *O. oeni* population decreases progressively, but it can negatively affect the wine quality by removing desirable aromas or by producing undesirable compounds such as harmful biogenic amines, mousy off-flavor, or bitterness (Bartowsky 2009).

Many studies have been carried out to unravel the diversity of *O. oeni* strains during wine production. There are always many strains in the fermenting grape must, but a selection occurs during the course of AF. On average, 2 to 6 strains are present during MLF, but not necessarily during all of the MLF because there may be a succession of strains from the beginning to the end (Reguant & Bordons 2003; Cappello et al. 2008; Mesas et al. 2011; Gonzalez-Arenzana et al. 2012b; El Khoury et al. 2017). The type of wine and winemaking practices modulates not only the LAB species and population, but also the strains of *O. oeni* (Gonzalez-Arenzana et al. 2013). A remarkable example is the presence of strains belonging to two different genetic lineages which preferentially develop in the French white wines of Burgundy and Champagne or in the red wines of Burgundy (Campbell-Sills et al. 2017). The main difference of the two lineages is that they tolerate better the low pH of white wines or in contrast phenolic compounds in red wines (Breniaux et al. 2018). However, it would be simplistic to consider that there is a strain type for each wine type. Even in the previous example, the strains of the two genetic lineages were isolated from wines in which other strains belonging to different genetic lineages were present (El Khoury et al. 2017).

## Vineyard and cellar: the origin of wine strains

Wine is a seasonal environment that permits the development of microorganisms only for a few months a year. The *O. oeni* strains that develop in wine originate from the surface of grapes in the vineyard, or from the cellar where they can persist by producing exopolysaccharides and biofilms at the surface of tanks, barrels, and other cellar’s equipment (Dimopoulou et al. 2014; Bastard et al. 2016). Nevertheless, *O. oeni* is a minor species in the oenological environment as soon as it is not in wine. It was not isolated from the vineyard (Bae et al. 2006; Yanagida et al. 2008), except in a recent study in which several strains were isolated from grapes of the Priorat region (Catalonia, Spain) (Franquès et al. 2017). For the first time, this study describes the same strains on grape and in wine, thus confirming the role of the vineyard as a source of strains that colonize wine. The role of the cellar’s equipment has not been directly established, but it is possible to detect commercial strains in cellars where they have been used in the past, suggesting that they were present in the cellar or its immediate environment (Gonzalez-Arenzana et al. 2014a; El Khoury et al. 2017; Franquès et al. 2017). The same “wild” strains are sometimes detected in wines of the same cellar during several consecutive vintages, but this does not indicate whether they are residents of the vineyard or the cellar (Reguant & Bordons 2003; Franquès et al. 2017).

## Apple cider: the second home

Apple cider is also a suitable environment for *O. oeni*. This is not very surprising given that cider and wine are close in terms of production process (AF and MLF), microbial diversity (yeasts and LAB), and composition (low pH, presence of ethanol, phenolic compounds, malic acid, etc.) (Cousin et al. 2017). *O. oeni* is one of the main LAB contributing to MLF in cider. It has always been detected along with other LAB species (Salih et al. 1988; Sánchez et al. 2010, 2012; Dierings et al. 2013). This contrasts with its predominance in wine, probably because cider has lower alcohol content (1.2–8%) and sometimes a higher pH than wine, which makes it more suitable for the growth of non-*O. oeni* species. The microbial biodiversity of cider is still incompletely described and, given the wide variety of ciders produced around the world, it is possible that *O. oeni* is absent in some of them, or on the opposite, predominant. Interestingly, cider and wine are two different environments that not only influence the biodiversity of LAB species, but also *O. oeni* strains. As discussed below, strains that preferentially develop in wine or in cider belong to different genetic lineages (El Khoury et al. 2017).

## Other natural habitats

While the presence of *O. oeni* in wine and cider is well documented, it has recently been identified as the main LAB species of a third fermented beverage (Coton et al. 2017). Kombucha is a traditional Asian drink that has become popular and industrially produced in North America and Europe. It is obtained by spontaneous fermentation of sweetened black or green tea by an indigenous microbiota composed of yeasts, acetic acid bacteria, and LAB. During fermentation, the pH drops down to 3.5–3.3 with the production of organic acids, and traces of alcohol may be produced (up to 1%). In a recent analysis of industrial production of French kombucha, *O. oeni* was not only detected in all fermentation tanks, but it was also the main LAB species (~ 10^5^ cfu mL^−1^) (Coton et al. 2017). It is clear that this environment is as favorable as wine and cider for *O. oeni*, although it remains to be determined which parameters, in addition to the low pH, can benefit to *O. oeni*. In addition, as mentioned above for cider and wine strains, those isolated from kombucha form to a distinct phylogenetic lineage, which suggests a specific adaptation of the species to this product (Lorentzen et al. in review).

*O. oeni* may be a minor species in other fermented beverages such as Brazilian kefir in which it was detected (Zanirati et al. 2015). It may be part of the natural microbiota that develops on rotting fruits or in fruit juices, such as mango juice (Ethiraj & Suresh 1985) or stone fruits (Bridier et al. 2010) from which it has been isolated, but its presence is most likely sporadic and minor. Nevertheless, all fermented products that might be appropriate for *O. oeni* have not yet been investigated. The recent examples of kombucha, but also Shochu for *O. kitaharae* and Cachaça for *O. alcoholitolerans*, suggest that it is still possible to identify new products that *O. oeni* has colonized.

## *O. oeni* strains diversity: methods and applications

Since the first description of the species in 1967, numerous studies have investigated the biodiversity *of O. oeni* strains in wine regions, vineyards, cellars, wines, ciders, and more recently kombucha. The first methods were used to differentiate strains by producing molecular fingerprints. This includes pulsed-field gel electrophoresis of large DNA fragments produced by restriction enzyme digestion of the bacterial chromosome (REA-PFGE). It was first used in 1993, and often afterwards, although it is difficult and time-consuming (Kelly et al. 1993; Tenreiro et al. 1994; Sato et al. 2001; Guerrini et al. 2003; López et al. 2007; Larisika et al. 2008; Gonzalez-Arenzana et al. 2012a, b; Zapparoli et al. 2012; Wang et al. 2015; Vigentini et al. 2016). More simple and rapid methods based on the use of PCR were later developed and applied, such as Rapid Amplification of Polymorphic DNA (RAPD) (Zavaleta et al. 1997; Zapparoli et al. 2000; Reguant & Bordons 2003; Lechiancole et al. 2006; Canas et al. 2009; Capozzi et al. 2010; Solieri et al. 2010; Marques et al. 2011), Amplified Fragment Length Polymorphism (AFLP) (Viti et al. 1996; Sato et al. 2000; Cappello et al. 2008; Cappello et al. 2010), or more recently Multiple Loci VNTR Analysis (MLVA), which targets genomic regions conserved among all strains but with different sizes as they contain a variable number of tandem repeats (VNTR) (Claisse & Lonvaud-Funel 2012; Claisse & Lonvaud-Funel 2014; Garofalo et al. 2015; Cruz-Pio et al. 2017; El Khoury et al. 2017; Franquès et al. 2017). The methods have revealed that there is a great diversity of strains in each region, several strains in each wine tank, and generally 2 to 6 major strains during MLF; that strains present in the vineyard at the surface of grapes contribute to MLF in wines produced from these grapes; that strains can persist in cellars for several years and thus contribute to MLF in wines produced during several consecutive vintages. They were also employed for assessing the biodiversity of cider strains (Sanchez et al. 2012), and they are still used today because they are simple, cost-efficient, and useful for analyzing large collections of strains or isolates. Nevertheless, these methods fail at providing data on the species population structure and phylogenetic proximity of the strains. Multi Locus Sequence Typing (MLST), which is based on the sequence analysis of housekeeping genes, was developed and used for this purpose (de Las Rivas et al. 2004; Bilhere et al. 2009; Bridier et al. 2010; Bordas et al. 2013; Gonzalez-Arenzana et al. 2014b; Wang et al. 2015; Romero et al. 2018). It has provided the first hints on the species population structure, showing that strains form at least two main genetic lineages, named groups A and B, and their incidence in regions and products. But nowadays, the method of choice is genome sequencing and comparative genomics. Since the first genome of strain PSU-1 produced in 2005 by Sanger technology (Mills et al. 2005), Next Generation Sequencing (NGS) technologies have made it possible to compare genomic sequences of 14 strains in 2012 (Borneman et al. 2012a), 57 in 2015 (Campbell-Sills et al. 2015), 196 in 2016 (Sternes & Borneman 2016), and more than 220 genomes are now available in databanks. Phylogenomics analyses have confirmed the population structure and phylogenetic lineages previously suggested by MLST. They have also revealed new strains lineages and allowed the discovery of some correlations with the regions or products of origin. Comparative genomics investigations have started to unravel the genetic characteristics of the strains, shedding new light on their adaptation to different environments.

## Diversity of strains in wine and other products

The first population structures revealed by MLST and phylogenomics analyzes of numerous strains isolated from diverse sources suggested that all the strains fall within the two groups A and B, except one strain which was tentatively attributed to a third group C (Bilhere et al. 2009; Bridier et al. 2010; Campbell-Sills et al. 2015; Sternes & Borneman 2016). Recently, adding new genomes of strains isolated from cider and kombucha to the 196 genomes analyzed previously has confirmed this third group C and revealed a fourth group D (Lorentzen et al. in review). Group A contains only wine strains (Fig. 1). Groups B and C contain both cider and wine strains. Group D only contains the 5 kombucha strains sequenced to date. This distribution suggests that there is a correlation between the phylogenetic groups and the products. Group A strains would be the most domesticated to wine because not only does this group contain exclusively wine strains, but almost all strains marketed to date belong to this group. In addition, as described previously, group A contains subgroups of strains that are even more domesticated to certain types of wine, such as white wines of Burgundy or Champagne (Campbell-Sills et al. 2017). The mixed compositions of groups B and C have long been puzzling. First, it is rare to isolate group B strains from wine. For example, they were not detected in 65 wines collected during MLF and analyzed by a PCR test targeting groups A or B (Campbell-Sills et al. 2015). Second, although group C contains wine strains, they have all been isolated from Australian wines, which could be explained by a regional specificity or by a specific sampling method that benefits to these strains. The solution was probably reached with the development of quantitative PCR tests for each group A-D (Lorentzen, unpublished). When they are used to monitor the populations of each group at different stages of wine production, it appears that strains of groups A, B, and C are present at similar levels in the grape must, whereas groups B and C strains disappear during AF, leaving only group A strains at the onset of MLF (Lorentzen, unpublished). It is likely that the different phylogenetic groups of *O. oeni* strains have evolved by adapting to different fermented beverages, kombucha, cider, and wine, as the close species *O. kitaharae* and *O. alcoholitolerans* have adapted to the fermentations of Shochu and sugar cane. Group A strains are best suited to develop in wine after AF and strains that belong to subgroups of A may be further adapted to specific types of wine. As mentioned above, adaptation to red and white wines was experimentally confirmed for group A strains that belong to the two subgroups named AW and AR (Campbell-Sills et al. 2017). The AW strains develop well at very low pH, whereas they are highly sensitive to phenolic compounds. On the opposite, AR strains are less tolerant to acidity, but more resistant to phenolic compounds (Breniaux et al. 2018). It is clear that AW and AR strains have evolved independently to further adapt to the two different types of wines: respectively the acidic white wines of Burgundy and Champagne, and the red wines of Burgundy.

## Diversity of strains in regions and the concept of microbial terroir

The geographical distribution of microorganisms is a major issue in the context of wine production, for which the quality and typicity of wine are strongly associated with the characteristics of the region of production, commonly grouped under the concept of terroir. Recently, NGS technologies have allowed accurately establishing the species abundance in the vineyard and in wine of different regions. They revealed that the grape microbial biodiversity is non-randomly associated with regions, climate, and grape variety, raising the concept of “microbial terroir” for describing microbial communities typical of wine production areas (Bokulich et al. 2014; Knight & Goddard 2015; Pinto et al. 2015). In addition, correlations have been made between the grape microbiota (yeasts or bacteria) and the presence in wine of specific metabolites that influence the quality perception (Knight et al. 2015; Bokulich et al. 2016). Although NGS approaches have revealed the relative abundance of *O. oeni* in the vineyard and at different stages of wine production, they give no insights on the prevalence of each strain, which is a major limitation in the description of the so-called microbial terroir because the quality of wine varies with the metabolic capacity of the fermenting strains (Stefanini & Cavalieri 2018). Nevertheless, the regional diversity of *O. oeni* strains is an unresolved issue. It is clear that each region contains a multitude of strains that belong to different genetic lineages, at least those of groups A and B, and probably also group C (El Khoury et al. 2017). This suggests that strains are not genetically adapted to regions, although we cannot exclude that some specific climatic conditions may benefit to some specific groups of strains. However, it is more likely that strains are adapted to the products they ferment rather than the regions where they survive when they are not in wine. For example, the subgroup of A strains which are well adapted to ferment the low pH white wines produced in Burgundy and Champagne is more linked to this type of wine than to each of these regions. It is likely that strains of this subgroup will be isolated from acidic white wines produced in other regions.

## Concluding remarks

Despite its inability to dominate other species in the natural environment, *O. oeni* has been able to become the main species in wine and one of the most important in cider, kombucha, and probably other fermented beverages. It is possible that all three species of the genus *Oenococcus* initially had a better ability than other LAB to grow in alcohol-containing environments. Nevertheless, it is obvious that they have recently evolved to become the best-adapted to their preferred man-made environments. The domestication of *O. oeni* is particularly evident at the intra-species level, where strains from different genetic lineages are better adapted to develop in different types of wines. The hypermutability linked to the absence of the MutSL system is undoubtedly a key factor in the rapid evolution and adaptation of the strains, but several points remain to be determined: the genetic changes associated to this adaptation to wine and to different types of wines, and why *O. oeni* has outperformed other species in this harsh environment.
